# Rolling Leaf 2 Controls Leaf Rolling by Regulating Adaxial-Side Bulliform Cell Number and Size in Rice

**DOI:** 10.3390/plants14213373

**Published:** 2025-11-04

**Authors:** Yu-Jia Leng, Shi-Yu Qiang, Wen-Yu Zhou, Shuai Lu, Tao Tao, Hao-Cheng Zhang, Wen-Xiang Cui, Ya-Fan Zheng, Hong-Bo Liu, Qing-Qing Yang, Ming-Qiu Zhang, Zhi-Di Yang, Fu-Xiang Xu, Hai-Dong Huan, Xu Wei, Xiu-Ling Cai, Su-Kui Jin, Ji-Ping Gao

**Affiliations:** 1 Jiangsu Key Laboratory of Crop Genomics and Molecular Breeding, Zhongshan Biological Breeding Laboratory, Key Laboratory of Plant Functional Genomics of the Ministry of Educations, Agricultural College of Yangzhou University, Yangzhou 225009, China; yujialeng@yzu.edu.cn (Y.-J.L.);; 2 Jiangsu Co-Innovation Center for Modern Industrial Technology of Grain Crops, Jiangsu Key Laboratory of Crop Genetics and Physiology, Yangzhou University, Yangzhou 225009, China; 3 Yangzhou Modern Seed Innovation Institute, Gaoyou 225600, China; 4 Joint International Research Laboratory of Agriculture and Agri-Product Safety, the Ministry of Education of China, Yangzhou University, Yangzhou 225009, China

**Keywords:** rice, rolling leaf, bulliform cells, genetic analysis, map-based cloning, *RLL2*

## Abstract

Leaves represent an important organ in plant photosynthesis, and moderately rolled leaves would be beneficial in establishing an ideal plant architecture and thereby increasing rice yields. In this study, a stable inherited rolled leaf mutant was obtained via ethyl methanesulfonate (EMS) mutagenesis from *japonica* variety WYJ27, which was named *rll2* (*rolling leaf 2*). *rll2* showed a leaf-rolling phenotype at the seedling stage, which increased with growth. Compared with the wild type, the leaves at all levels of *rll2* were significantly shorter and narrower, and the leaf-rolling index gradually decreased from the highest leaf to the third-highest leaf. Semi-thin sections showed that the bulliform cells of *rll2* were significantly larger than those of the wild type, and the number of cells was significantly higher than that of the wild type. Genetic analysis showed that *rll2* is controlled by a pair of recessive nuclear genes. Map-based cloning revealed that *RLL2* encodes a conserved and plant-specific calpain-like cysteine proteinase. *RLL2* was mainly expressed in young roots, shoots, spikelets, and panicles. Transcriptome sequencing showed that a total of 104 genes were differentially expressed in the wild type and *rll2*. Moreover, several transcription factor genes were significantly altered in the *rll2* mutant. Taken together, our findings indicate that *RLL2* plays an important role in leaf rolling by regulating bulliform cells, which may be useful in breeding rice with an ideal plant architecture.

## 1. Introduction

Leaves represent the main site of photosynthesis in plants and provide energy for plant growth. As an important part of plant architecture, leaf morphology is closely related to rice yields [1]. Moderate leaf rolling not only contributes to improving light acceptance, delaying leaf senescence, and accelerating the accumulation of dry matter but also helps to improve root system activity and strengthen lodging resistance [2,3]. Therefore, the study of leaf rolling has high practical value in high-yield rice breeding.

Leaf development is a complex process comprising cell division and expansion, axis determination, and tissue differentiation and specification [4]. In rice, leaf development involves three stages: the initiation of leaf primordia, polarity establishment and maintenance, and leaf flattening and intercalary growth [5,6]. The leaf primordium originates from the peripheral zone of the apical shoot meristem (SAM) [7], develops along the adaxial–abaxial, medial–lateral, and apical–basal polarity axes, and finally forms mature leaves [8,9]. This developmental process is regulated by complex regulatory networks involving numerous functional genes, transcription factors, phytohormones, and microRNAs [1,3]. To date, many genes controlling leaf rolling have been identified in rice, such as *SHALLOT-LIKE1*/*ROLLED LEAF 9* (*SLL1*/*RL9*) [10,11], *SHALLOT-LIKE1* (*SLL2*) [12], *SEMI-ROLLED LEAF 1* (*SRL1*) [13,14], *SEMI-ROLLED LEAF 1* (*SRL2*) [2], *ABAXIALLY CURLED LEAF 1* (*ACL1*) [15], *ABAXIALLY CURLED LEAF 2* (*ACL2*) [15], *NARROW AND ROLLED LEAF 1* (*NRL1*) [16], *NARROW LEAF 7* (*NAL7*) [17], *Rolling-leaf14* (*RL14*) [18], *ADAXIALIZED LEAF 1* (*ADL1*) [19], *ARGONAUTE 1b* (*AGO1b*) [20], *Rolled and Erect Leaf 1* (*REL1*) [21], *lateral organ boundaries domain* (*LBD*) gene *LBD3-7* [22], zinc finger homeodomain class homeobox gene *OsZHD1* [23], class IV homeodomain gene *CFL1* [24], class III HD-Zip gene *OsHOX32* [25], *RICE OUTERMOST CELLSPECIFIC GENE* (*Roc*) family gene *Roc5* [26], and *Roc8* [27]. Most of these genes regulate leaf rolling by changing the morphology of leaf bulliform cells, sclerenchyma cells, cells in vascular bundles, cuticle, epidermal cells, and mesophyll cells.

Bulliform cells, also called hygroscopic cells or motor cells, are among the most important cell structures and are found on the adaxial epidermis of the leaf. A change in osmotic pressure in bulliform cells can cause leaf rolling. Advances in research have suggested that leaf rolling is primarily caused by bulliform cell changes in rice [21]. For example, *SLL1* encodes a SHAQKYF-class MYB family transcription factor. The *sll1* mutant shows a leaf-rolling phenotype due to the formation of bulliform cells on the abaxial epidermis [10]. *SRL1* encodes a putative glycosylphosphatidylinositol-anchored protein, which causes adaxial rolling in leaves by inhibiting the formation of bulliform cells [13]. *REL1* encodes a high-degree conservation and unknown-function protein in monocot plants. The *rel1* mutant shows a leaf rolling due to an increase in the size and number of bulliform cells [21]. Although many genes relating to leaf rolling have been identified, the molecular mechanisms behind leaf rolling in rice are not yet fully understood. In the present study, we identified and characterized a rolled-leaf mutant, named *rolling leaf 2* (*rll2*), from a *japonica* variety Wuyunjing27 (WYJ27) EMS mutant library. Using a map-based cloning method, we cloned the *RLL2* gene and found that it encodes a conserved and plant-specific calpain-like cysteine proteinase, allelic to the *ADL1* gene. The loss of *RLL2* function leads to an increase in the number and size of bulliform cells on the adaxial side of the leaf, resulting in abaxially rolled leaves. This study enhances our understanding of the role of *RLL2*/*ADL1* in leaf rolling.

## 2. Materials and Methods

### 2.1. Plant Materials

The rice *rll2* mutant was obtained from ethyl methanesulfonate-treated (EMS) *japonica* cultivar Wuyunjing27 (WYJ27). An F_2_ segregation population was obtained by crossing *rll2* with an *indica* cultivar IR36. WYJ27, IR36, *rll2*, and F_2_ segregation populations were germinated and sown on Yangzhou University’s experimental farms in Lingshui (110°00′ E, 18°31′ N) and Yangzhou (119′ 42 E, 32°39′ N) during the rice-growing season. After 30 days, the seedlings were transplanted into a field at a density of 18 cm × 18 cm. About 150 kg N ha^−1^ (as urea), 130 kg K_2_O ha^−1^, and 60 kg P_2_O_5_ ha^−1^ were added to each field. Field management, including irrigation and pest control, followed normal agricultural practices.

### 2.2. Measurement of Leaf-Rolling Index and Agronomic Traits

The leaf-rolling index (LRI) was calculated using the following formula: LRI = (Lw − Ln)/Lw × 100% [28]. Lw was defined as the largest leaf width in the fully expanded leaves; Ln was defined as the natural distance of the leaf margins. The plant height (PH), tiller number (TN), primary branch number (PBN), secondary branch number (SBN), and grain number per panicle (GNPP) were measured at 25 days after heading. The plant height was measured from the base to the tip of the tallest panicle. The panicle at the highest point on the plant was selected to investigate the grain number. Except for two marginal plants on each side, twenty independent plants were randomly selected to score the phenotypic data. The rice grains were harvested at 35 days after flowering, dried in the sun, and then transferred to a room-temperature environment. Once the physical and chemical properties were stable, the grain shape traits, including grain length (GL), grain width (GW), and thousand-grain weight (TGW), were determined using the WSeen SC-G automatic seed testing system and thousand-grain weight analysis system (Hangzhou WSeen Detection Technology Co., Ltd., Hangzhou, China). About 150–200 grains were evaluated. Grain thickness (GT) was measured using a digital vernier caliper. A total of ten grains were used to measure GT.

### 2.3. Histology and Microscopic Observation

Hand-cut sections of WT and *rll2* leaves (about 35 days after sowing) were observed on a slide with an OLYMPUS SZX16 stereomicroscope (Olympus Corporation, Tokyo, Japan). To observe leaf anatomy in detail, young leaves were fixed in a formalin–acetic acid–alcohol (FAA) solution (50% ethanol, 5% formaldehyde, 5% glacial acetic acid = 18:1:1 *v*:*v*:*v*) at 4 °C overnight. After dehydration in serial concentrations of ethanol (50%, 70%, 90%, 100%, 100%), they were infiltrated and embedded in Technovit 7100 resin (Heraeus Kulzer, Hanau, Germany). Sectioning (3 μm) was conducted with the Leica RM2265 (Leica, Wetzlar, Germany). Slices were spread on a platform at 42 °C and stained using toluidine blue at room temperature for 15 min. Observations and photography were carried out using the Leica DM2500 LED light microscope (Leica, Wetzlar, Germany).

### 2.4. Genetic Analysis and Map-Based Cloning of RLL2

An F_2_ mapping population was derived from a cross between *rll2* and IR36. A total of 859 individuals with the mutant phenotype were used for fine mapping. The *rll2* loucs was first mapped to an interval between markers M1 and M2 on chromosome 2. Further, it was narrowed to a 225 kb region between maker M6 and M7. The molecular markers were developed with Primer Premier 5 software (Premier Biosoft International, Palo Alto, CA, USA) based on sequence differences between the *japonica* variety Nipponbare and the *indica* variety 93-11. The molecular markers used in this study are listed in Table S1. Gene prediction and sequence analysis were performed using the Rice Genome Annotation Project (http://rice.uga.edu/index.shtml (accessed on 3 September 2023)) databases. For the identification of the candidate gene, the corresponding DNA fragments were amplified from WYJ27 and *rll2* using Kod FX (TOYOBO, Osaka, Japan) and sequencing using Applied Biosystems 3730xl DNA Analyzers (Applied Biosystems, Carlsbad, CA, USA). The primers for sequencing in this study are listed in Table S2.

### 2.5. Phylogenetic Analysis

The amino acid sequences of the RLL2 homological proteins were retrieved by searching the NCBI BLAST database (https://blast.ncbi.nlm.nih.gov/Blast.cgi (accessed on 10 June 2024)) using the total amino acid sequences of RLL2. Phylogenetic trees were constructed with the aligned protein sequences using MEGA 12 software based on the neighbor-joining statistical method with the following parameters: Poisson model, uniform rate, pairwise deletion, and bootstrap (1000 replicates) [29].

### 2.6. RNA Extraction and qRT-PCR

The total RNA of various tissues in WYJ27 and *rll2* was extracted using the RNAprep Pure Plant kit (TIANGEN, Beijing, China), and reverse transcription into cDNA was conducted with the PrimeScript™ RT reagent kit with gDNA Eraser (TaKaRa, Shiga, Japan) according to the manufacturer’s instructions. The cDNA was used for real-time RT-PCR using the TB Green^®^ Premix EX Taq^TM^ kit (TaKaRa, Shiga, Japan) and gene-specific primers (Table S3) with a CFX Connect^TM^ Real-Time System (Bio-Rad Laboratories, Hercules, CA, USA). The rice *ACTIN* gene was used as an internal standard to normalize the expression level of *RLL2* and other tested genes. The relative expression levels were calculated based on the comparative Ct method using the 2^−∆∆Ct^ formula [30].

### 2.7. RNA-Seq Analysis

Twenty days after sowing, the flat leaves of wild-type WYJ27 and the rolled leaves of the mutant *rll2* were selected for transcriptome sequencing, and each sample was pooled for total RNA isolation with three biological replicates. Total RNA was extracted using the RNAprep Pure Plant kit (TIANGEN, Beijing, China) according to the manufacturer’s protocol. Transcriptome sequencing was performed by Biomarker Technologies Corporation using the Illumina HiSeq2500 system (Illumina, San Diego, CA, USA). Transcriptome assembly was performed according to the protocol described previously [31]. In brief, the raw RNA-Seq data were processed using in-house Perl scripts to trim adapters and low-quality sequences and then assembled using Trinity based on left.fq and right.fq [32]. Differential expression analysis was performed using the DESeq R package (version 1.40.0). The fold change (FC) ≥ 2 and false discovery rate (FDR) < 0.01 were used as the screening criteria. Gene function was annotated based on the KEGG (Kyoto Encyclopedia of Genes and Genomes) and GO (Gene Ontology) databases. GO and KEGG pathway enrichment analysis was performed using BMKCloud (www.biocloud.net).

### 2.8. Statistical Analysis

Data are presented as the mean ± standard deviations of multiple biological replicates (*n* ≥ 3). Significant differences were assessed using Student’s *t* test. *p* < 0.05 and *p* < 0.01 were defined as significant and highly significant, respectively.

## 3. Results

### 3.1. Phenotypic Characterization of the rll2 Mutant

**Figure 1:**
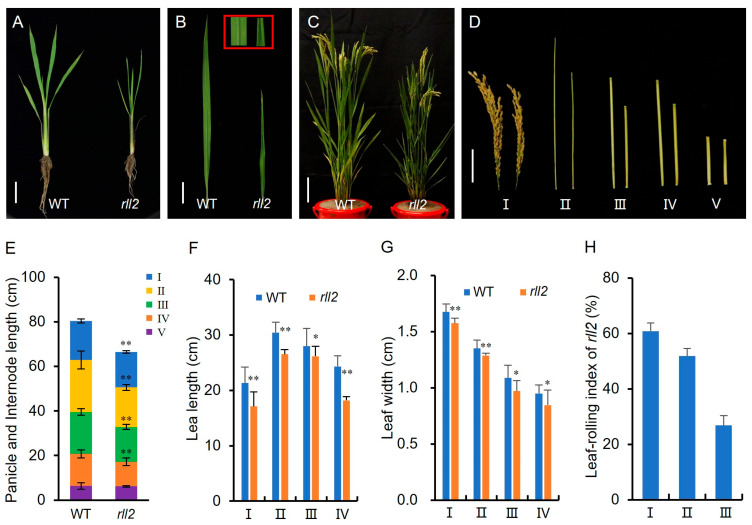
Comparison of wild-type and *rll2* phenotypes. (**A**) Phenotype of the wild type (WYJ27, left) and *rll2* mutant (right) at 35 days after sowing. Scale bar = 5 cm. (**B**) Difference in leaf phenotype between the wild type (left) and *rll2* (right) mutant 35 days after sowing. Scale bar = 2 cm. (**C**) Phenotype of the wild type (WYJ27, left) and *rll2* mutant (right) 10 days after heading. Scale bar = 5 cm. (**D**) Phenotype of panicles and internodes between the wild type (WYJ27, left) and *rll2* mutant (right) at the mature stage. Scale bar = 5 cm. I: panicle, II: highest internodes, III: second-highest internodes, IV: third-highest internodes, V: fourth-highest internodes. (**E**) Statistical analysis of panicle and internode length between the wild type and the *rll2* mutant at the mature stage. F and G. Statistical analysis of leaf length (**F**) and leaf width (**G**) 10 days after heading. (**H**) Leaf-rolling index of the *rll2* mutant 10 days after heading. I: Highest leaf, II: second-highest leaf, III: third-highest leaf, IV: fourth-highest leaf. * Significant difference at *p* < 0.05 determined via *t*-test; ** Significant difference at *p* < 0.01 determined via *t*-test.

The *rll2* mutant was identified by screening an ethyl methanesulfonate (EMS) mutant library for the ‘Wuyunjing 27’ (WYJ27) background and had abaxially rolled leaves throughout the entire growth period (Figure 1A–C). The leaf-rolling index (LRI) of *rll2* at the heading stage from the highest (flag leaf) to the third-highest leaf was 60.9%, 51.9%, and 26.9%, respectively, whereas wild-type WYJ27 leaves were nearly flat (Figure 1H). *rll2* also displayed short and narrow leaves; the length and width of the highest, second-highest, third-highest, and fourth-highest leaves in *rll2* were 80.1%, 87.1%, 93.6%, and 74.8% and 94.0%, 94.5%, 89.2%, and 89.0% of those for the wild type, respectively (Figure 1F,G). Furthermore, the yield traits including plant height, internode length, tillering number, primary branch number, secondary branch number, grain number per panicle, grain length, grain width, and thousand-grain weight in *rll2* were significantly lower than those of the wild type (Figure 1D,E; Table 1). Taken together, these results indicate that *RLL2* has pleiotropic effects in rice growth and development.

### 3.2. Bulliform Cell Number and Size Are Increased in rll2

**Figure 2:**
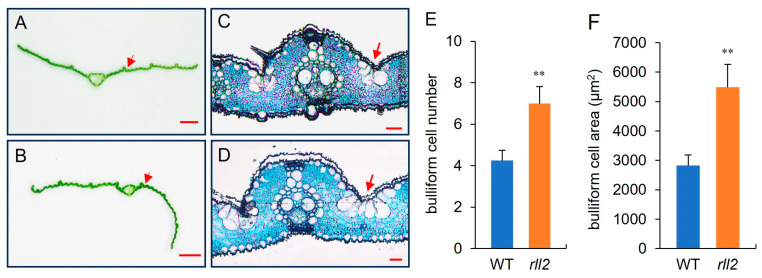
*rll2* shows an increase in bulliform cell number and size. (**A**,**B**). Cross-section of wild-type (**A**) and *rll2* (**B**) leaves 35 days after sowing. WYJ27, scale bar = 2 mm. (**C**,**D**). Semi-thin section of wild-type (**C**) and *rll2* (**D**) leaves 35 days after sowing. WYJ27, scale bar = 100 μm. The red arrow indicates bulliform cells. (**E**,**F**). The numbers (**E**) and areas (**F**) of bulliform cells in WYJ27 and *rll2*. Data are shown as means ± standard deviation. ** Significant difference at *p* < 0.01 determined via *t*-test.

To investigate the cause of abaxial leaf rolling in *rll2*, we analyzed cross-sections of leaves f at 35 days after sowing (Figure 2A–D). In contrast to the wild type, more bulliform cells occurred between two vascular bundle ridges in the adaxially leaf. The bulliform cell number for *rll2* was 7.0 ± 0.8 cells, which was significantly higher than that of the wild type (4.3 ± 0.5) (Figure 2E). Furthermore, the bulliform cell area of *rll2* was also significantly higher than that of the wild type (Figure 2F). Those results suggest that the abaxial leaf rolling in *rll2* may be caused by an increase in bulliform cell number and size on the adaxial surface of leaves.

### 3.3. Map-Based Cloning of RLL2

**Figure 3:**
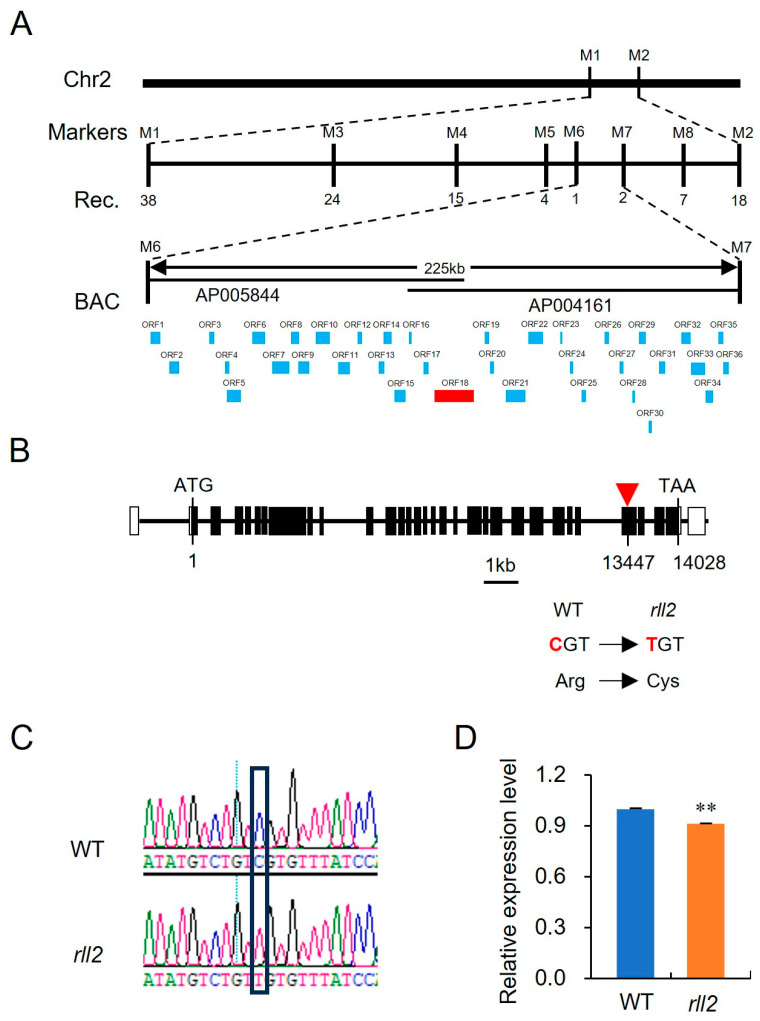
Map-based cloning of *RLL2*. (**A**) Fine mapping of *RLL2*. The *RLL2* locus was mapped to a 225 kb region on chromosome 2. (**B**) Schematic diagram of *RLL2*. Black rectangles represent exons. White rectangles represent 5′ and 3′-UTR. The red inverted triangle represents the mutant site. (**C**) Sequencing analysis of ORF18 between WYJ27 and *rll2.* (**D**) The expression levels of LOC_Os02g47970 in WYJ27 and the *rll2* mutant. Each sample comprised three independent experiments. ** *p* < 0.01.

To isolate *RLL2* via map-based cloning, we crossed the *rll2* mutant with an *indica* cultivar, IR36. All the F_1_ hybrids exhibited a WT morphological phenotype. In the F_2_ segregating populations, the WT morphological phenotype and rolled-leaf phenotype showed a typical segregation ratio of 3:1 (Table 2). This result indicates that *rll2* is controlled by a single recessive nuclear gene. The *RLL2* locus was preliminarily mapped to the chromosome 2 between markers M1 and M2 (Figure 3A). By using a large population of 859 homozygous mutant individuals, *RLL2* was narrowed down to a 225 kb genomic region between markers M6 and M7 on BAC clone AP004161 (Figure 3A). According to the annotations of the rice genome database (Rice Genome Annotation Project, https://rice.uga.edu/), this region comprises twenty putative genes with annotated functions. A known gene controlling leaf rolling, *ADL1* (*LOC_Os02g47970*), was identified in these ORFs. The *adl1* mutant showed abaxially rolled leaves due to an increase in the size and number of bulliform cells. The *rll2* mutant displayed a similar phenotype with *adl1*. DNA sequencing analysis of this ORF revealed the presence of a single nucleotide substitution (C→T) on the 27th exon of *LOC_Os02g47970* in *rll2* compared to the wild type, which resulted in an Arg-to-Cys substitution (Figure 3B,C). Moreover, the expression level of *LOC_Os02g47970* in *rll2* was significantly decreased (Figure 3D). We therefore inferred that *ADL1* was the gene controlling the *rll2* mutant phenotype.

### 3.4. RLL2 Encodes a Conserved and Plant-Specific Protein

**Figure 4:**
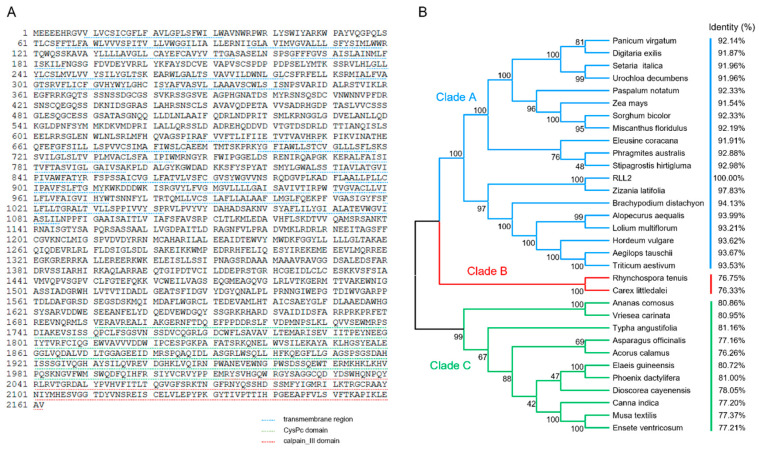
Amino acid sequence and phylogenetic analysis of RLL2. (**A**) Amino acid sequence of RLL2. The blue dotted line, red dotted line, and green dotted line represent the transmembrane region, CysPc domain, and calpain_III domain, respectively. (**B**) Phylogenetic relationships among RLL2-like proteins.

Sequence analysis indicated that *RLL2* cDNA is 6489 bp in length and encodes a protein of 2162 amino acid residues, including 22 transmembrane regions, 1 CysPc domain, and 1 calpain_III domain (Figure 4A). We performed a BLASTP search against the NCBI non-redundant protein database, revealing 31 proteins from other plant species with significant homology (≥70% identity at the amino acid level) to RLL2, which suggests that RLL2 is a plant-specific protein and shares conserved biochemical functions. To elucidate the evolutionary relationships among RLL2 proteins, a phylogenetic tree was constructed using the neighbor-joining method in MEGA 12 [29]. The 32 proteins were classified into three clades (Figure 4B). Clade A belongs to Gramineae and shows high identity (>90%) with the RLL2 protein. The identity of clade B and clade C with the RLL2 protein was lower than that of clade A, which ranged from 76.26% to 81.16%. This result indicates that the RLL2 protein is very conserved in evolution.

### 3.5. Expression Patterns of RLL2

**Figure 5:**
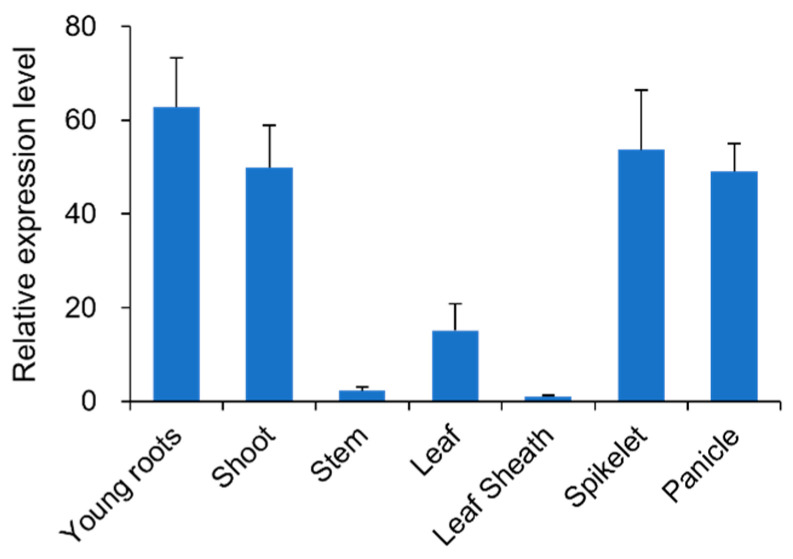
Expression pattern of *RLL2*. Each sample comprised three independent experiments.

To determine the expression patterns of *RLL2* in rice, quantitative real-time RT-PCR (qRT-PCR) was used to detect the expression levels of *RLL2* in WYJ27 issues, such as young roots, shoots, stems, leaves, leaf sheaths, spikelets, and panicles. The results showed that *RLL2* was expressed in all tissues, with high expression in young roots, shoots, spikelet and panicles and low expression in stems and leaf sheaths (Figure 5).

### 3.6. GO and KEGG Analyses of Rolling Leaf Regulation via RLL2

**Figure 6:**
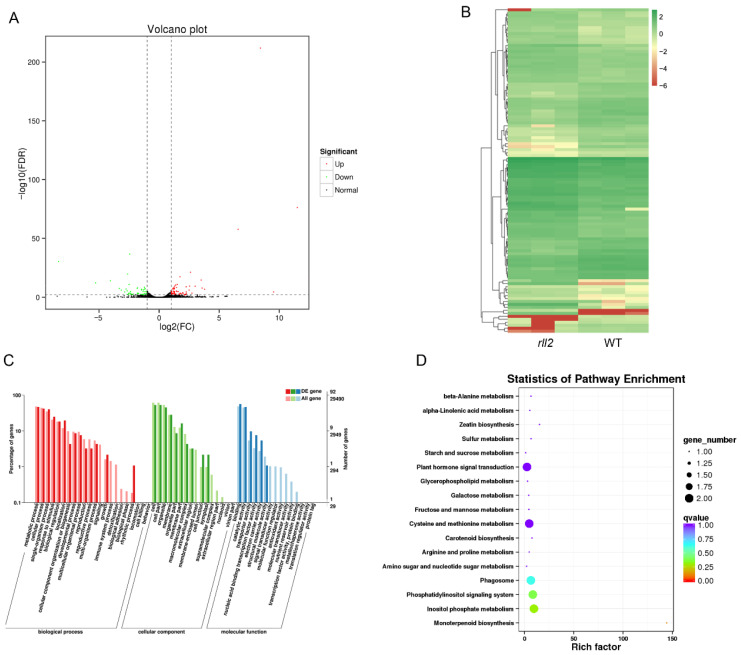
Transcriptome analysis of the wild type and *rll2*. (**A**) Volcano map of differentially expressed genes between the wild type and *rll2*. (**B**) Cluster diagram of differentially expressed genes between the wild type and *rll2*. (**C**) GO enrichment analysis for the wild type and *rll2*. (**D**) KEGG pathway enrichment analysis for the wild type and *rll2*.

To further unravel the function of *RLL2* in rice, leaves from wild-type and *rll2* plants at 20 d were used for transcriptome analysis. A total of 104 differentially expressed genes (DEGs) were found; 42 genes were up-regulated and 62 were down-regulated in *rll2* plants (Figure 6A,B; Table S5). Among these genes, 12 transcription factor genes were found, 10 of which have been reported. We measured the expression levels of these genes using qRT-PCR, and the results showed that the expression levels of three genes (*OsbHLH056*, *OsDREB1B*, and *OsWRKY71*) were significantly increased and those of seven genes (*OsDREB1A*, *OsAP2-39*, *PCF2*, *OsERF74*, *OsNAC5*, *OsNAC19*, and *LOC_Os05g50340*) were significantly reduced in *rll2* leaves (Figure S1; Table S5).

The GO enrichment analysis of DEGs is shown in Figure 6C and Table S6. In the cellular component analysis, DEGs were found to mainly be involved in the cell, cell part, organelle, membrane, and membrane part. In the molecular function analysis, DEGs were found to mainly be involved in binding, catalytic activity, transporter activity, and nucleic acid binding transcription factor activity. In the biological process analysis, DEGs were found to mainly be involved in the metabolic process, cellular process, single-organism process, response to stimulus, localization, and biological regulation. KEGG pathway enrichment analysis showed that the DEGs were mainly concentrated in plant hormone signal transduction, cysteine and methionine metabolism, phagosome, inositol phosphate metabolism, and the phosphatidylinositol signaling system (Figure 6D).

### 3.7. RLL2 Affects the Expression Levels of Leaf Development-Related Genes

**Figure 7:**
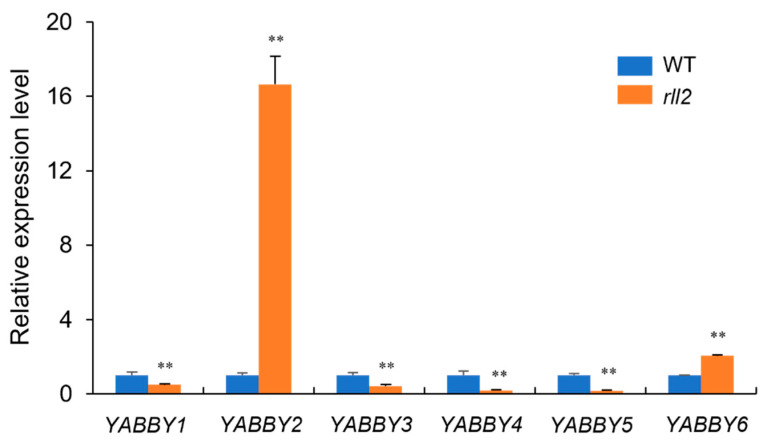
Expression levels of *YABBY* genes in the wild type and *rll2*. Each sample comprised three independent experiments. ** Significant difference at *p* < 0.01 determined via *t*-test.

Leaf development is regulated by several genes, such as *YABBY* genes [33,34,35,36]. We measured the expression levels of *YABBY* genes in young leaves via qRT-PCR. Compared with the wild type, *YABBY2* and *YABBY6* were significantly up-regulated in *rll2*, while *YABBY1*, *YABBY3*, *YABBY4*, and *YABBY5* were significantly down-regulated in *rll2* (Figure 7). This result suggests that *rll2* phenotypes may be related to altered transcriptional activity of *YABBY* genes.

## 4. Discussion

### 4.1. RLL2 Plays an Important Role in Regulating the Size and Number of Leaf Bulliform Cells in Rice

Bulliform cells (motor cells), which are distributed on the upper epidermis of leaves, are the most important cell structures in leaves [27]. Bulliform cells are strongly linked to leaf-rolling phenotypes in rice [37]. Many studies have shown that alternations to bulliform cells arranged on the adaxial surface of the leaf lead to adaxial or abaxial rolling in mature leaves [21]. To date, over 10 genes controlling bulliform cell development have been reported in rice; *ACL1* [15], *ACL2* [15], *NRL1* [16], *NAL7* [17], and *RL14* [18] positively regulate the development of bulliform cells, while *SRL1* [13,14], *ADL1* [19], *OsLBD3-7* [22], *OsZHD1* [23], and *Roc5* [26] negatively regulate the development of bulliform cells. In this study, we identified *RLL2* as a key regulator of leaf rolling. Compared to the wild type, *rll2* showed a higher number and size of bulliform cells, leading to abaxial leaf rolling (Figure 2). Our results strongly suggest that *RLL2* plays a negative role in the formation of bulliform cells on the adaxial leaf surface.

### 4.2. RLL2 Is a New Allelic Variant of ADL1

Leaves are the main photosynthetic organs in plants. Moderate leaf rolling is considered to be an important part of the ideal rice phenotype, the development of which is of great significance in improving photosynthetic efficiency and grain yields [2,10,38]. However, the molecular mechanisms underlying leaf rolling remain unclear. In this study, we identified the *rll2* mutant from an EMS-mutagenized *japonica* variety, WYJ27 (Figure 1). Using a map-based cloning method, gene sequencing, and qRT-PCR, we found that the candidate gene is *ADL1*/*LOC_Os02g47970*, which encodes calpain-like cysteine proteinase (Figure 3 and Figure 4). Hibara et al. (2009) reported seven *ADL1* allelic mutants (*adl1-1*, *adl1-2*, *adl1-3*, *adl1-s1*, *adl1-s2*, *adl1-g1*, and *adl1-g2*), which produced a base mutation in exon 30, 26, 4, 26, 19, 27, and 20, respectively, resulting in changes in the amino acids encoded [19]. In our study, we identified an *RLL2* mutation on the 27th exon, with a C to T change (Figure 3B,C), indicating that *RLL2* is a new allelic mutant of *ADL1* [19]. The *rll2* mutant showed a rolling leaf phenotype similar to that of the *adl1* mutant, but there were some phenotypic differences among the *rll2*/*adl1* mutant alleles; for example, the *adl1* mutant showed bulliform cells on the abaxial side of leaves, while the mutant *rll2* did not. *adl1-1*, *adl1-2*, and *adl1-3* showed defects in leaf polarity and leaf primordium insertion in the SAM. *adl1-s1* and *adl1-s2* produced shootless embryos. The embryos of *adl1-g1* and *adl1-g2* stopped growing at the globular stage, and the endosperm lacked a ventral aleurone layer [19]. This indicates that different mutant *RLL2*/*ADL1* alleles have different effects on rice development. In addition, KEGG pathway enrichment analysis showed that cysteine and methionine metabolism genes differed between the wild type and *rll2* (Figure 6D). These changes may impact the function of calpain-like cysteine proteinase in *rll2*. Further investigation is needed to fully understand the role of *RLL2* in rice.

### 4.3. Transcription Factor Genes Participate in the Development of Leaves in RLL2

Transcription factors (TFs) are a group of DNA-binding proteins that regulate gene expression and are involved in a diverse range of biological processes in plants [39]. In rice, several transcription factors have been reported as being involved in the regulation of leaf rolling; For example, *SLL1* encodes an SHAQKYF-class MYB transcription factor belonging to the KANADI family. It regulates leaf rolling in rice by regulating the development of cells on the abaxial side of the leaf [10]. *Roc8* is a member of the plant-specific homeodomain leucine zipper IV (HD-Zip IV) gene family, encoding a class of homeobox transcription factors. The overexpression of *Roc8* causes leaf rolling on the adaxial side, and its knockout causes leaf rolling on the abaxial side [27]. *OsMYB103L* encodes an R2R3-MYB transcription factor. The overexpression of *OsMYB103L* leads to a rolled-leaf phenotype. Further analysis shows that *OsMYB103L* regulates cellulose synthesis by targeting *CESA* genes, thereby affecting leaf rolling [40]. In our study, the expression levels of 10 transcription factor genes changed in 104 DEGs, indicating that transcription factors play an important role in leaf rolling (Figure S1; Table S5).

*YABBY* genes are plant-specific transcription factors that play an important role in the regulation of a diverse range of developmental processes, such as the establishment of adaxial–abaxial polarity, laminal expansion, and floral organ development [36,41,42]. Many *YABBY* genes have been studied in monocotyledon plants, and several *YABBY* genes have been cloned in rice. *OsYABBY1* shows homology with *AtYAB2* and *AtYAB5*, is expressed in sclerenchyma, and participates in the development of the stamen, carpel, and meristem [43]. *YAB3* is closely related to maize *ZmYAB14* and *Arabidopsis FILAMENTOUS FLOWER* (*FIL*), which is mainly related to the development of leaves [33]. *OsYABBY4* is involved in the development of the vascular structure [44]. *OsYABBY5* (*TOB1*) plays a vital role in promoting lateral organ development and maintaining meristem organization in the rice spikelet [36]. *DROOPING LEAF* (*DL*), a rice orthologue of CRC, is mainly related to carpel specification and midvein development in rice [45]. In our study, the expression level of *YABBY* genes in the *rll2* mutant was altered. *YABBY2* and *YABBY6* were significantly up-regulated in *rll2*, while *YABBY1*, *YABBY3*, *YABBY4*, and *YABBY5* were significantly down-regulated. In particular, the expression of *YABBY2* was significantly up-regulated (16-fold) in *rll2* (Figure 7). This suggests that the *rll2* phenotype may be related to altered transcriptional activity in *YABBY* genes.

Rice leaf morphology is an important component in achieving the ideal plant architecture and significantly impacts rice yields [1]. Using mutagenesis techniques to identify mutants with moderately rolled leaves is beneficial in breeding crops with the desired architecture [2]. Although our study shows that *RLL2* plays an important role in regulating leaf rolling, more research is needed on, for example, the effect of *RLL2* gene mutation on calpain-like cysteine proteinase activity, how transcription factors genes affect the development of bulliform cells by regulating *RLL2*, and whether certain *RLL2* alleles are preponderant in the improvement of rice leaf morphology. These questions require further study.

## 5. Conclusions

The *rolling leaf 2* (*rll2*) mutant was obtained through the ethyl methanesulfonate (EMS) mutagenesis of the *japonica* variety WYJ27. The *rll2* mutant displayed abaxially rolled leaves throughout the entire growth period. We demonstrated that *rll2* is a new allele of *ADL1*, which encodes a CysPc domain and calpain_III domain protein. We suggest that the increase in the number and size of bulliform cells is causative of leaf rolling in the *rll2* mutant. Moreover, qRT-PCR showed that the expression of some transcription factor genes changed significantly in *rll2*. Our study provides a novel allelic mutant to explore the function of *RLL2*. At the same time, it provides useful information for furthering the improvement of rice architecture.

## Figures and Tables

**Table 1:** Comparison of major agronomic traits between the wild type and *rll2*.

Traits	Wild Type	*rll2*
Plant height (cm)	80.4 ± 5.56	66.5 ± 1.02 **
Tillering number	9.6 ± 1.19	9.0 ± 0.76 **
Primary branch number	11.8 ± 1.16	8.6 ± 0.92 **
Secondary branch number	33.0 ± 2.00	24.6 ± 4.93 **
Grain number per panicle	182.0 ± 12.76	135.8 ± 23.36 **
Grain length (mm)	7.6 ± 0.37	7.4 ± 0.28 **
Grain width (mm)	3.5 ± 0.30	3.4 ± 0.30 **
Grain thickness (mm)	2.4 ± 0.03	2.4 ± 0.02
Thousand-grain weight (g)	28.6 ± 0.46	26.1 ± 0.36 **

** Significant difference at *p* < 0.01 determined via *t*-test.

**Table 2:** Genetic analysis of *rll2*.

Cross Combination	F_1_		F_2_		χ2 (3:1)
	Wild-Type Phenotype	*rll2* Phenotype	Wild-Type Phenotype	*rll2* Phenotype	
*rll2*/IR36	36	0	483	169	0.2945
IR36/*rll2*	28	0	363	109	0.9153

## Data Availability

The original contributions presented in this study are included in the article/Supplementary Material. Further inquiries can be directed to the corresponding authors.

## Supplementary Materials

The following supporting information can be downloaded at: https://www.mdpi.com/article/10.3390/plants14213373/s1, Figure S1. Expression levels of twelve transcription factor genes in wild type and *rll2*; Table S1. The primers used for gene mapping; Table S2. The primer used for *RLL2* gene sequencing; Table S3. The primer used for qRT-PCR; Table S4. The candidate gene for *RLL2* between marker M6 and M7; Table S5. Differentially expressed gene in wild type and *rll2*; Table S6. GO enrichment analysis of differentially expressed genes in *rll2*. References [46,47,48,49,50,51,52,53,54,55,56,57,58,59,60,61,62,63,64,65,66,67,68,69,70,71,72,73,74,75,76,77,78,79,80,81] are cited in the Supplementary Materials.
