# Supplementary material for: Rolling Leaf 2 Controls Leaf Rolling by Regulating Adaxial-Side Bulliform Cell Number and Size in Rice
Source: Plants (Basel). 2025 Nov 4;14(21):3373. doi: 10.3390/plants14213373 (PMC12608271; doi:10.3390/plants14213373)
Supplement: Supplementary file 1 [file plants-14-03373-s001.zip › plants-3837316-supplementary.pdf]

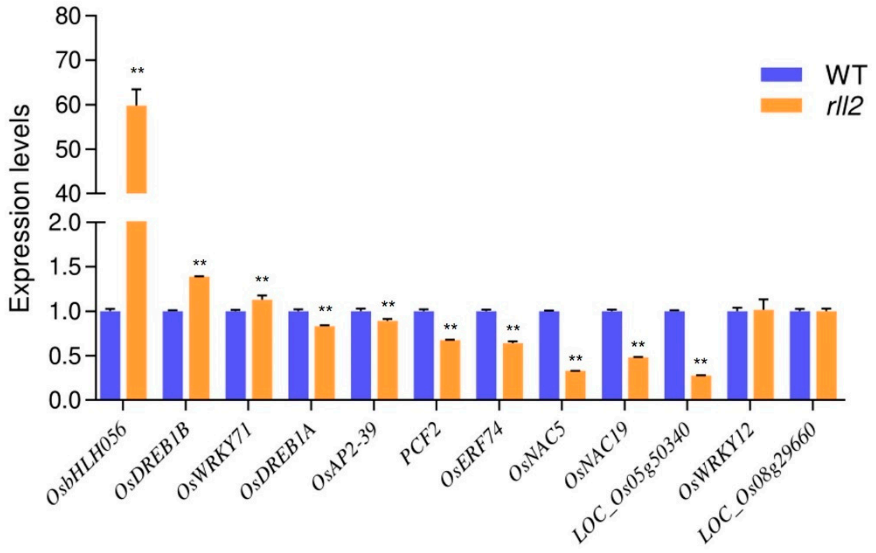

Figure S1. Expression levels of twelve transcription factor genes in wild type and *rll2*.

Table S1. The primers used for gene mapping.

Primer name	Forward primer sequence	Reverse primer sequence
M1	GGAAAGAAGATATGCCATGC	AGAGAGAATCAGAAACACCAAC
M2	TCACCACCGTCATCCTCAAC	AGCCGCAAACCGATCACAAT
M3	GTTCCCGTATCATCAACGAG	CTGATCGAGCGGTGTAGC
M4	TCTCTGAACACACCCACACC	TGTTTTGATCTCAGCTCCCC
M5	CCACAACACCAACGATGCTA	TAGGACTGACCCCTCTGCAT
M6	GCATAGTTGAACCAGTAGCATG	GAACTGGCAGAATTGAGTGTC
M7	GATACTGATACTCCAAGAAGGCACT	TAATGATAGACATGACGCAAACC
M8	TTCGACGATTCTGTACACGC	CATGATCCCAAATGCATGGG

Table S2. The primer used for *RLL2* gene sequencing.

Primer name	Forward primer sequence	Reverse primer sequence
RL-1	TGCCTGCATACTTAGTGTCATTT	AATCCTCCGTGAAGCTTTAAA
RL-2	ACTTCTAAGTCATGGGTTCA	AACACAGGCCCAAATTCCAGTC
RL-3	CGTGCTTGTTCTATTATGCTGTT	CTACATCTCCTAGTCCACCATTC
RL-4	TAGATAGTGGACGTGCCAGTTTA	CAAGGCAAAATGCAGTAGTAACTAA
RL-5	GTTCTGTCCTCCTTAACTCGTCT	GTGGCGACTAGGATGTGGAG
RL-6	GAAAACTCTTACTTGATCCCCTT	TCAGCATCCCATCCCTTGTAGCC
RL-7	ATAGTGTAGAACGCATTCAGACC	GAAACTATTATGTAACTCGGAGA
RL-8	TGTTACTTGTTTGCTCCCTTG	CAAGCAAGAACAGCATTTACCT
RL-9	AGCCGCTCACATCAGTGGCACAT	TTTAGATGGCGGGCACAGAGGTT
RL-10	CAGCATAGGTCTTTCTGACTTAA	TTGTCATCCTTTCACCCTTAGTC
RL-11	TGCAGTAATGGTGCAGCCGGT	TGTCCATCCCATTCAACGTCCTC
RL-12	TTTCGACCAATCTATTCCTACTG	TGCTCTACCAATATACTTTACCA
RL-13	TGTTCTTTTCAGGGTCGGTTG	CACAGGACAATCTCAAGTCTTTTTT
RL-14	CATGAGTGAACAAGTCACTAATG	CTTCATTCGCTCAGTCCACTCT
RL-15	CAGGTTTGCTCTGACTTGTCT	AACCCCAAAGGAGCCTGGGCCAG

Table S3. The primer used for qRT-PCR.

Primer name	Forward primer sequence	Reverse primer sequence
YABBY1	TGGTGAATGTGCCAAACAAT	GCTTGGTGAAAAGGAGCAAG
YABBY2	AATTTTCGCGGTCAGTGTTC	AGTTTTCGCGGAAGCTCATA
YABBY3	ATCAAGGACGAAATCCAACG	GGCATCAGTCCAAAGTGGAT
YABBY4	ATCACATCAAAGGGGACCAA	CGAGTACGCAATGGCCTTAT
YABBY5	GAGCCTAATGACCGAACAGG	CTCTGCCGCTTCTCTGAAGT
YABBY6	TCTGTTCATCACTGGCTTCG	CGTGTTGCAGAAGTTGCAGT
qRLL2	GCTGGCTGGGTGCTTTAACTTC	ACACAGGCCCAAATTCCAGTCG
OsbHLH056	AGCTCAACGAGCTCTACTCCTC	GTCGGGATGCTCAGCTTCTTAG
OsAP2-39	TCGTCCGTTTAATTGCCAGGATTG	ACTGTAACACCGGGAGTAGTAGCC
PCF2	ATTACTCCCGCGTGTTGTGCTG	AGCTAAGGACGGGAAAGTAAGGAG
OsWRKY12	ACACGAGGTGATGATCTCTTTCCC	GCTCTTCTTGCTGCTCCTTCTC
OsWRKY71	CAAGCCCAAGATCTCCAAGCTC	TTGGTACCCATCTTTCACCACCAG
OsNAC5-F	TCAGGTTGGATGATTGGGTGTTG	TCGTACCTCTCGATCACTCCCTTC
OsNAC19	GCCAAGAAGGGATCTCTCAGGTTG	TCTTCTTGTTGTACAGCCGACAC
OsERF74	CGCCGGCGATGTAGTTTAAGAG	ACGCCGGCAGGATATGTCTTAG
OsDREB1A	AGCCGTCCTCGTGTAGAAACAG	TCGTCACTGTAGTTCTCGTGCAG
OsDREB1B	AGAGAGTCATCCATGGAGGTGGAG	TCGTCTCCCTGAACTTGGTCCTTC
LOC_Os05g50340	ACGTCAAGCGCTACTACGAGATG	TTCAACCTTTCGGCCTGGTAGC
LOC_Os08g29660	TGAAGGGCGAGATATATGGTCCAC	CATGGCCTATCTCCTCTAAACAGC
Actin	CGGAGCGTGGTTACTCATTCA	TCCAGGGCGATGTAGGAAAG

Table S4. The candidate gene for *RLL2* between marker M6 and M7.

Number	Gene ID	Putative Function
1	LOC_Os02g47800	Monodehydroascorbate reductase
2	LOC_Os02g47810	dof zine finger domain containing protein,
3	LOC_Os02g47820	expressed protein
4	LOC_Os02g47830	expressed protein
5	LOC_Os02g47840	universal stress protein domain containing protein
6	LOC_Os02g47850	class I glutamine amidotransferase
7	LOC_Os02g47860	uridine kinase
8	LOC_Os02g47870	anaphase-promoting complex subunit 11
9	LOC_Os02g47880	tetratricopeptide repeat domain containing protein
10	LOC_Os02g47890	expressed protein
11	LOC_Os02g47900	SET domain protein SOG117
12	LOC_Os02g47910	hypothetical protein
13	LOC_Os02g47920	ZOS2-16 - C2H2 zinc finger protein
14	LOC_Os02g47930	expressed protein
15	LOC_Os02g47940	aminotransferase, classes I and II, domain containing protein
16	LOC_Os02g47950	expressed protein
17	LOC_Os02g47960	expressed protein
18	LOC_Os02g47970	calpain
19	LOC_Os02g47980	DUF617 domain containing protein,
20	LOC_Os02g47990	retrotransposon protein
21	LOC_Os02g48000	TBC domain containing protein,
22	LOC_Os02g48010	nuclear matrix constituent protein 1-like,
23	LOC_Os02g48020	expressed protein
24	LOC_Os02g48030	expressed protein
25	LOC_Os02g48040	expressed protein
26	LOC_Os02g48050	hypothetical protein
27	LOC_Os02g48060	helix-loop-helix DNA-binding domain containing protein
28	LOC_Os02g48070	expressed protein
29	LOC_Os02g48080	cysteine-rich receptor-like protein kinase 7 precursor
30	LOC_Os02g48090	expressed protein
31	LOC_Os02g48094	expressed protein
32	LOC_Os02g48100	DEAD-box ATP-dependent RNA helicase
33	LOC_Os02g48110	DnaK family protein
34	LOC_Os02g48116	expressed protein
35	LOC_Os02g48122	PPR repeat domain containing protein
36	LOC_Os02g48130	uncharacterized protein At1g26090, chloroplast precursor

Table S5. Differentially expressed gene in wild type and *rll2.*

Up or down-regulated	Differentially expressed gene	FPKM	Putative Function	Reference
UP-regulated	Os04g0571600	3.3585	MATE efflux family protein	
	Os01g0952800 (*OsIRO2*/*OsbHLH056*)	4.0152	bHLH transcription factor	[1, 2]
	Os03g0152000	3.2443	heavy-metal-associated domain-containing protein	
	Os05g0495700	2.6019	glycerol-3-phosphate dehydrogenase	
	Os01g0699600 (*OsMKKK62*)	3.4217	Mitogen activated protein kinase kinase kinase	[3]
	Os02g0667100	3.7768	expressed protein	
	Os03g0767000 (*OsAOS1*)	2.5213	cytochrome P450	[4]
	Os02g0661100 (*OsTPP1*)	2.7263	trehalose-6-phosphate phosphatase	[5]
	Os10g0195250	2.5482	expressed protein	
	Os01g0597600 (*OsATL15*)	4.9513	ransmembrane amino acid transporter protein	[6]
	Os01g0699500 (*OsMKKK70*)	5.0501	Mitogen activated protein kinase kinase kinase	[7]
	Os02g0703600 (*OsABA8ox*)	3.3676	ABA 8′-Hydroxylase	[8]
	Os04g0610400 (*OsAP2-39*)	2.8055	APETALA-2-Like transcription factor	[9]
	Os08g0544800 (*PCF2*)	2.3190	TCP family transcription factor	[10]
	Os03g0823301	4.7590	transporter, major facilitator superfamily domain containing protein	
	Os06g0318533	4.3732	expressed protein	
	Os06g0203000	5.5141	Hypothetical protein	
	Os09g0522200 (*OsDREB1A*/*OsCBF3*)	5.1225	AP2/EREBP transcription factor	[11, 12]
	Os10g0391400 (*OsJAZ13*)	10.0184	ZIM domain containing protein	[13]
	Os11g0151400	16.3236	cytochrome P450	
	Os05g0305200	72.4853	TRAF-type zinc finger family protein	
	Os06g0137700	592.4118	GDP-mannose 4,6 dehydratase 2	
	Os11g0570000 (*COG1*)	6.1456	leucine-rich repeat receptor like protein	[14]
	Os01g0624700 (*OsWRKY12*)	6.2928	WRKY transcription factor	[15]
	Os09g0442100	2.0218	tyrosine protein kinase domain containing protein	
	Os02g0181300 (*OsWRKY71*)	3.1517	WRKY transcription factor	[16]
	Os07g0258400 (*OsNRAMP1*)	2.2499	metal transporter Nramp6	[17]
	Os08g0386200	2.1617	WRKY transcription factor	
	Os01g0834900	5.5426	Hypothetical conserved gene	
	Os03g0109900	5.0291	expressed protein	
	Os03g0815100 (*OsNAC19*)	2.6831	NAC domain-containing protein 67	[18]
	Os01g0647200 (*OsIMA1*)	2.4387	Iron deficiency-inducible peptide	[19]
	Os05g0497300 (*OsERF74*)	2.1668	AP2/ERF transcription factor	[20]
	Os11g0184900 (*OsNAC5*)	2.2506	NAC domain transcription factor	[21]
	Os03g0181100 (*OsJAZ10*)	5.9864	ZIM domain containing protein	[22]
	Os02g0584800	1.9728	heavy metal associated domain containing protein	

	Os07g0142100 (*OsIMA2*)	2.4600	Iron deficiency-inducible peptide	[19]
	Os12g0181600	2.2839	amino acid transporter	
	Os10g0469000	6.3581	leucine-rich repeat receptor protein kinase EXS precursor	
	Os04g0481550	5.8760	Hypothetical gene	
	Os09g0522000 (*OsDREB1B*)	4.9849	DREB1/CBF-type transcription factor	[23]
	Os06g0292400	6.5011	cadmium tolerance factor	
down-regulated	Os05g0582000	0.2441	secretory protein	
	Os10g0112700	0.1671	OsWAK98 - OsWAK receptor-like cytoplasmic kinase OsWAK-RLCK	
	Os06g0663100	0.3163	tRNA pseudouridine synthase family protein	
	Os02g0755900 (*OsUGT3*)	0.3041	UDP-glycosyltransferase	[24]
	Os12g0493900	0.2832	pumilio-family RNA binding repeat domain containing protein	
	Os04g0556400	0.2171	glucosyltransferase	
	Os07g0561300 (*OsFBX257*)	0.2266	OsFBX257 - F-box containing protein	[25]
	Os08g0360300	0.1233	calmodulin binding protein	
	Os01g0959100 (*Asr1*)	0.2002	abscisic acid-stress-ripening-inducible 1 protein	[26]
	Os01g0225300	0.1561	expressed protein	
	Os04g0691900	0.1990	phosphatidylinositol-4-phosphate 5-Kinase	
	Os12g0235200	0.2501	expressed protein	
	Os02g0258800	0.2029	expressed protein	
	Os11g0489250	0.2760	cytochrome P450	
	Os01g0359400	0.5240	disease resistance protein RGA4	
	Os04g0607500 (*OsHKT1;1*)	0.4027	high-affinity potassium transporter	[27]
	Os02g0245800 (*OsKAT3*)	0.4737	potassium channel KAT1	[28]
	Os01g0800500	0.4900	nucleotide pyrophosphatase/phosphodiesterase	
	Os09g0484900	0.4510	citrate transporter	
	Os06g0220100	0.3082	Hypothetical protein	
	Os12g0503000 (*OsUPS1*)	0.3979	ureide permease	[29]
	Os10g0130800	0.5197	disease resistance RPP13-like protein 1	
	Os01g0856000	0.4876	Putative DNA replication initiation protein	
	Os04g0171800	0.3576	cytochrome P450	
	Os05g0202800	0.4075	metallothionein-like protein 3B	
	Os05g0217700	0.3435	expressed protein	
	Os04g0683700 (*OsAAE3*)	0.3707	AMP-binding domain containing protein	[30]
	Os04g0671300 (*OsPAO5*)	0.4149	amine oxidase, flavin-containing, domain containing protein	[31]
	Os08g0503700	0.4821	citrate transporter	
	Os03g0843800 (*OsHOL1*)	0.4463	methyltransferase	[32]
	Os09g0451400 (*OsACO1*)	0.4308	1-aminocyclopropane-1-carboxylate oxidase protein	[33]
	Os04g0380300	0.4902	OsFBK14 - F-box domain and kelch repeat containing protein	
	Os01g0854000	0.4868	transferase family protein	

Os04g0531750	0.3988	short-chain dehydrogenase/reductase	
Os09g0451266	0.4759	Hypothetical gene	
Os05g0579600	0.4726	MYB family transcription factor	
Os07g0209100	0.4025	uncharacterized glycosyltransferase	
Os03g0261100 (*PLA2-II*)	0.4064	phospholipases A2	[34]
Os03g0273800 (*OsHAD3*)	0.3748	haloacid dehalogenase-like hydrolase family protein	[35]
Os05g0217800	0.2134	BURP domain containing protein	
Os07g0509800	0.3050	OsAPRL1 adenosine 5'-phosphosulfate reductase-like OsAPRL1	
Os03g0345700	0.1583	heavy metal-associated domain containing protein	
Os06g0220000	0.3580	phosphate-induced protein 1 conserved region domain containing protein	
Os07g0198300	0.4310	LTPL84 - Protease inhibitor/seed storage/LTP family protein precursor	
Os02g0636600	0.2898	GEM	
Os10g0509401	0.3249	UP-9A	
Os03g0257600	0.3968	DUF292 domain containing protein	
Os10g0542400	0.4203	expansin precursor	
Os08g0480000	0.3848	MATE efflux family protein	
Os01g0342500	0.3865	expressed protein	
Os09g0478300	0.3516	CSLE6 - cellulose synthase-like family E	
Os08g0450700	0.0008	chaperonin Cpn60/TCP-1	
Os12g0219700	0.0095	expressed protein	
Os01g0385400 (*SLAC7*)	0.2104	C4-dicarboxylate transporter/malic acid transport protein	[36]
Os03g0200000	0.1370	haemolysin-III	
Os10g0159300	0.2107	retrotransposon	
Os01g0622033	0.0788	Hypothetical gene	
Os03g0711425	0.0547	Hypothetical gene	
Os06g0178650	0.2027	iron/ascorbate-dependent oxidoreductase	
Os02g0697500	0.0028	pentatricopeptide	
Os07g0236300	0.0863	Hypothetical gene	
Os06g0699050	0.0000	Hypothetical gene	

References

[1] Ogo, Y.; Itai, R.N.; Nakanishi, H.; Inoue, H.; Kobayashi, T.; Suzuki, M.; Takahashi, M.; Mori, S.; Nishizawa, N.K. Isolation and characterization of IRO2, a novel iron-regulated bHLH transcription factor in graminaceous plants. *J. Exp. Bot.* **2006**, 57, 2867–2878.

[2] Wang, S.; Li, L.; Ying, Y.; Wang, J.; Shao, J.F.; Yamaji, N.; Whelan, J.; Ma, J.F.; Shou, H. A transcription factor OsbHLH156 regulates Strategy II iron acquisition through localising IRO2 to the nucleus in rice. *New Phytol.* **2020**, 225, 1247–1260.

[3] Mao, X.; Zhang, J.; Liu, W.; Yan, S.; Liu, Q.; Fu, H.; Zhao, J.; Huang, W.; Dong, J.; Zhang, S.; et al. The MKKK62-MKK3-MAPK7/14 module negatively regulates seed dormancy in rice. *Rice*. **2019**, 12, 2.

[4] Haga, K.; Iino, M. Phytochrome-mediated transcriptional up-regulation of *ALLENE OXIDE SYNTHASE* in rice seedlings. *Plant Cell Physiol.* **2004**, 45, 119–128.

[5] Pramanik, M.H.R.; Imai, R. Functional identification of a trehalose 6-phosphate phosphatase gene that is involved in transient induction of trehalose biosynthesis during chilling stress in rice. *Plant Mol. Bio.* **2005**, 58, 751–762.

[6] Xiao, Y.;Zhang, H.; Li, Z.; Huang, T.; Akihiro, T; Xu, J.; Xu, H.; Lin, F. An amino acid transporter-like protein (OsATL15) facilitates the systematic distribution of thiamethoxam in rice for controlling the brown planthopper. *Plant Biotechnol. J.* **2022**, 20, 1888–1901.

[7] Liu, Z.; Mei, E.; Tian, X.; He, M.; Tang, J.; Xu, M.; Liu, J.; Song, L.; Li, X.; Wang, Z.; et al. OsMKKK70 regulates grain size and leaf angle in rice through the OsMKK4-OsMAPK6-OsWRKY53 signaling pathway. *J. Integr. Plant Biol.* **2021**, 63, 2043–2057.

[8] Yang, S.H.; Choi, D. Characterization of genes encoding ABA 8′-hydroxylase in ethylene-induced stem growth of deepwater rice (*Oryza sativa* L.). *Biochem. Biophys. Res. Commun.* **2006**, 350, 685–690.

[9] Yaish, M.W.; El-kereamy, A.; Zhu, T.; Beatty, P.H.; Good, A.G.; Bi, Y.M.; Rothstein, S.J. The APETALA-2-Like transcription factor OsAP2-39 controls key interactions between abscisic acid and gibberellin in Rice. *PLoS Genet.* **2010**, 6, e1001098.

[10] Kosugi, S.; Ohashi, Y. PCF1 and PCF2 specifically bind to *cis* elements in the rice proliferating cell nuclear antigen gene. *Plant Cell* **1997**, 9, 1607–1619.

[11] Dubouzet, J.G.; Sakuma, Y.; Ito, Y.; Kasuga, M.; Dubouzet, E.G.; Miura, S.; Seki, M.; Shinozaki, K.; Yamaguchi-Shinozaki, K. *OsDREB* genes in rice, *Oryza sativa* L., encode transcription activators that function in drought-, high-salt- and cold-responsive gene expression. *Plant J.* **2003**, 33, 751–763.

[12] Jia, M.; Meng, X.; Song, X.; Zhang, D.; Kou, L.; Zhang, J.; Jing, Y.; Liu, G.; Liu, H.; Huang, X.; et al. Chilling-induced phosphorylation of IPA1 by OsSAPK6 activates chilling tolerance responses in rice. *Cell Discov.* **2022**, 8, 71.

[13] Feng, X.; Zhang, L.; Wei, X.; Zhou, Y.; Dai, Y.; Zhu, Z. OsJAZ13 negatively regulates jasmonate signaling and activates hypersensitive cell death response in rice. *Int. J. Mol. Sci.* **2020**, 21, 4379.

[14] Xia, C.; Liang, G.; Chong, K.; Xu, Y. The COG1-OsSERL2 complex senses cold to trigger signaling network for chilling tolerance in japonica rice. *Nat. Commun.* **2023**, 14, 3104.

[15] Xie, Z.; Zhang, Z.L.; Zou, X.; Huang, J.; Ruas, P.; Thompson, D.; Shen, Q.J. Annotations and functional analyses of the rice *WRKY* gene superfamily reveal positive and negative regulators of abscisic acid signaling in aleurone cells. *Plant Physiol.* **2005**, 137, 176–189.

[16] Liu, X.; Bai, X.; Wang, X.; Chu, C. OsWRKY71, a rice transcription factor, is involved in rice defense response. *J. Plant Physiol.* **2007**, 164, 969–979.

[17] Takahashi, R.; Ishimaru, Y.; Nakanishi, H.; Nishizawa, N.K. Role of the iron transporter OsNRAMP1 in cadmium uptake and accumulation in rice. *Plant Signal. Behav.* **2011**, 6, 1813–1816.

[18] Lin, R.; Zhao, W.; Meng, X.; Wang, M.; Peng, Y. Rice gene *OsNAC19* encodes a novel NAC-domain transcription factor and responds to infection by *Magnaporthe grisea*. *Plant Sci.* **2007**, 172, 120–130.

[19] Kobayashi, T.; Nagano, A.J.; Nishizawa, N.K. Iron deficiency-inducible peptide-coding genes *OsIMA1* and *OsIMA2* positively regulate a major pathway of iron uptake and translocation in rice. *J. Exp. Bot.* **2021**, 72, 2196–2211.

[20] Fu, J.; Pei, W.; He, L.; Ma, B.; Tang, C.; Zhu, L.; Wang, L.; Zhong, Y.; Chen, G.; Wang, Q.; et al. ZmEREB92 plays a negative role in seed germination by regulating ethylene signaling and starch mobilization in maize. *PLoS Genet.* **2023**, 19, e1011052.

[21] Sperotto, R.A.; Ricachenevsky, F.K.; Duarte, G.L.; Boff, T.; Lopes, K.L.; Sperb, E.R.; Grusak, M.A.; Fett, J.P. Identification of up-regulated genes in flag leaves during rice grain filling and characterization of OsNAC5, a new ABA-dependent transcription factor. *Planta* **2009**, 230, 985–1002.

[22] Hakata, M.; Kuroda, M.; Ohsumi, A.; Hirose, T.; Nakamura, H.; Muramatsu, M.; Ichikawa, H.; Yamakawa, H. Overexpression of a rice TIFY gene increases grain size through enhanced accumulation of carbohydrates in the stem. *Biosci. Biotechnol. Biochem.* **2012**, 76, 2129–2134.

[23] Gutha, L.R.; Reddy, A.R. Rice *DREB1B* promoter shows distinct stress-specific responses, and the overexpression of cDNA in tobacco confers improved abiotic and biotic stress tolerance. *Plant Mol. Biol.* **2008**, 68, 533–555.

[24] Wang, T.; Ma, Y.Q.; Huang, X.X; Mu,T.J.; Li, Y.J.; Li, X.K.; Liu, X.; Hou, B.K. Overexpression of *OsUGT3* enhances drought and salt tolerance through modulating ABA synthesis and scavenging ROS in rice. *Environ. Exp. Bot.* **2021**, 192, 104653.

[25] Sharma, E.; Bhatnagar, A.; Bhaskar, A.; Majee, S.M.; Kieffer, M.; Kepinski, S.; Khurana, P.; Khurana, J.P. Stress-induced F-Box protein-coding gene *OsFBX257* modulates drought stress adaptations and ABA responses in rice. *Plant Cell Environ.* **2023**, 46, 1207–1231.

[26] Philippe, R.; Courtois, B.; McNally, K.L.; Mournet, P.; El-Malki, R.; Paslier, M.C.L.; Fabre, D.; Billot, C.; Brunel, D.; Glaszmann, J.C.; et al. Structure, allelic diversity and selection of Asr genes, candidate for drought tolerance, in *Oryza sativa* L. and wild relatives. *Theor. Appl. Genet.* **2010**, 121, 769–787.

[27] Wang, R.; Jing, W.; Xiao, L.; Jin, Y.; Shen, L.; Zhang, W. The rice high-affinity potassium transporter1;1 is involved in salt tolerance and regulated by an MYB-Type transcription factor. *Plant Physiol.* **2015**, 168, 1076–1090.

[28] Hwang, H.; oon, J.; Kim, H.Y.; Min, M.K.; Kim, J.A.; Choi, E.H.; Lan, W.Z.; Bae, Y.M.; Luan, S.; Cho, H.; et al. Unique features of two potassium channels, OsKAT2 and OsKAT3, expressed in rice guard cells. *PLoS ONE*, **2013**, 8(8): e72541.

[29] Lee, D.K.; Redillas, M.C.F.R.; Jung, H.; Choi, S.; Kim, Y.S.; Kim, J.K. A nitrogen molecular sensing system, comprised of the *ALLANTOINASE* and *UREIDE PERMEASE 1* genes, can be used to monitor N status in rice. *Front. Plant Sci.* **2018**, 9: 444.

[30] Liu, H.; Guo, Z.; Gu, F.; Ke, S.; Sun, D.; Dong, S.; Liu, W.; Huang, M.; Xiao, W.; Yang, G.; et al. 4-coumarate-CoA ligase-like gene *OsAAE3* negatively mediates the rice blast resistance, floret development and lignin biosynthesis. *Front. Plant Sci.* **2016**, 7, 2041.

[31] Lv, Y.; Shao, G.; Jiao, G.; Sheng, Z.; Xie, L.; Hu, S.; Tang, S.; Wei, X.; Hu, P. Targeted mutagenesis of *POLYAMINE OXIDASE 5* that negatively regulates mesocotyl elongation enables the generation of direct-seeding rice with improved grain yield. *Mol. Plant* **2021**, 14, 344–351.

[32] Carlessi, M.; Mariotti, L.; Giaume, F.; Fornara, F.; Perata, P.; Gonzali, S. Targeted knockout of the gene *OsHOL1* removes methyl iodide emissions from rice plants. *Sci. Rep.* **2021**, 11, 17010.

[33] Iwamoto, M.; Baba-Kasal, A.; Kiyota, S.; Hara, N.; Takano, M. *ACO1*, a gene for aminocyclopropane-1-carboxylate oxidase: effects on internode elongation at the heading stage in rice. *Plant Cell Environ.* **2010**, 33, 805–815.

[34] Kim, J.Y.; Chung, Y.S.; Ok, S.H.; Lee, S.G.; Chung, W.I.; Kim, I.Y.; Shin, J.S. Characterization of the full-length sequences of phospholipase A2 induced during flower development. *Biochim Biophys Acta.* **1999**, 1489, 389–392.

[35] Zan, X.; Zhou, Z.; Wan, J.; Chen, H.; Zhu, J.; Xu, H.; Zhang, J.; Li, X.; Gao, X.; Chen, R.; et al. Overexpression of *OsHAD3*, a member of HAD superfamily, decreases drought tolerance of rice. *Rice* **2023**, 16: 31.

[36] Fan, X.; Wu, J.; Chen, T.; Tie, W.; Chen, H.; Zhou, F.; Lin, Y. Loss-of-function mutation of rice SLAC7 decreases chloroplast stability and induces a photoprotection mechanism in rice. *J. Integr. Plant Biol.* **2015**, 57, 1063–1077.

Table S6. GO enrichment analysis of differentially expressed genes in *rll2*.

GO_classify1	GO_classify2	DEG number
Cellular component	extracellular region	3
	cell	49
	membrane	26
	cell junction	2
	macromolecular complex	4
	organelle	42
	organelle part	8
	membrane part	15
	cell part	49
	symplast	2
Molecular function	nucleic acid binding transcription factor activity	7
	catalytic activity	43
	structural molecule activity	1
	transporter activity	9
	binding	52
	electron carrier activity	5
Biological process	reproduction	3
	metabolic process	43
	cellular process	39
	reproductive process	3
	multicellular organismal process	7
	developmental process	8
	growth	2
	single-organism process	37
	rhythmic process	1
	response to stimulus	23
	localization	18
	multi-organism process	4
	biological regulation	17
	cellular component organization or biogenesis	4
